# Landscape of Variability in Chemosensory Genes Associated With Dietary Preferences in Indian Population: Analysis of 1029 Indian Genomes

**DOI:** 10.3389/fgene.2022.878134

**Published:** 2022-07-12

**Authors:** P. Prakrithi, Pankaj Jha, Jushta Jaiswal, Disha Sharma, Rahul C. Bhoyar, Abhinav Jain, Mohamed Imran, Vigneshwar Senthilvel, Mohit Kumar Divakar, Anushree Mishra, Vinod Scaria, Sridhar Sivasubbu, Mitali Mukerji

**Affiliations:** ^1^ CSIR- Institute of Genomics and Integrative Biology, Delhi, India; ^2^ Academy of Scientific and Innovative Research (AcSIR), Ghaziabad, India; ^3^ Department of Bioscience and Bioengineering, Indian Institute of Technology, Jodhpur, India

**Keywords:** chemosensory perceptions, genetic diversity, population differentiation, nutrigenomics, food preferences, precision nutrition

## Abstract

Perception and preferences for food and beverages determine dietary behaviour and health outcomes. Inherent differences in chemosensory genes, ethnicity, geo-climatic conditions, and sociocultural practices are other determinants. We aimed to study the variation landscape of chemosensory genes involved in perception of taste, texture, odour, temperature and burning sensations through analysis of 1,029 genomes of the IndiGen project and diverse continental populations. SNPs from 80 chemosensory genes were studied in whole genomes of 1,029 IndiGen samples and 2054 from the 1000 Genomes project. Population genetics approaches were used to infer ancestry of IndiGen individuals, gene divergence and extent of differentiation among studied populations. 137,760 SNPs including common and rare variants were identified in IndiGenomes with 62,950 novel (46%) and 48% shared with the 1,000 Genomes. Genes associated with olfaction harbored most SNPs followed by those associated with differences in perception of salt and pungent tastes. Across species, receptors for bitter taste were the most diverse compared to others. Three predominant ancestry groups within IndiGen were identified based on population structure analysis. We also identified 1,184 variants that exhibit differences in frequency of derived alleles and high population differentiation (F_ST_ ≥0.3) in Indian populations compared to European, East Asian and African populations. Examples include *ADCY10*, *TRPV1*, *RGS6*, *OR7D4*, *ITPR3*, *OPRM1*, *TCF7L2*, and *RUNX1*. This is a first of its kind of study on baseline variations in genes that could govern cuisine designs, dietary preferences and health outcomes. This would be of enormous utility in dietary recommendations for precision nutrition both at population and individual level.

## Introduction

Indian cuisines are extremely diverse across the country and reflect inherent differences in ethnic, geo-climatic, and socio-cultural practices. These could have co-evolved in populations with differences in perception of taste, odour, and flavour that guide dietary preferences and behaviour. As dietary behaviour impacts chronic and complex diseases, it becomes more important to understand the variability in chemosensory perception for precision nutrition recommendations. The ability to perceive distinct tastes (sweet, sour, pungent, bitter, salty, umami), flavour and texture has reported genetic underpinnings (Dotson et al., 2012). Variability in these genes is associated with dietary preferences, food intake behaviour and risk for diseases (Newcomb et al., 2012; Diószegi et al., 2019). For instance, individuals classified as “Supertasters,” “Moderate-Tasters” and “Non-Tasters” differ in their ability to perceive bitter tasting foods with supertasters having extreme sensitivity to very low thresholds due to variations in a bitter taste receptor gene *TAS2R38* (Duffy et al., 2004). Because of this, supertasters avoid alcohol, caffeine containing beverages and also healthy cruciferous vegetables and have higher preference for sweet foods. Similarly, variability in olfactory genes *OR7D4*, *OR11H7P*, *OR6A2* govern differences in preference for pork meat, cheesy food, and cilantro respectively (Menashe et al., 2003; Eriksson et al., 2012; Lunde et al., 2012). Variations in genes, for example, *CD36* also govern the ability to perceive textures of fatty food that impart flavour in food preparations (Keller et al., 2012). A significant number of chemosensory SNPs have also been associated with different non-metabolic diseases. For instance, the human bitter taste receptors (T2Rs) are implicated in chronic rhinosinusitis, asthma, thyroid levels, cystic fibrosis, and risk for cancer (Clark et al., 2015; Shaik et al., 2016). Similarly, disrupting the function of sodium channels such as the transient receptor potential cation subfamily V member 1 channel (*TRPV1*) and ENaC (*SCNN1B* gene) and variants associated with these genes modulate the perception for salt taste (Dias et al., 2013). A recent study of 2,854 SNPs in 50 taste-related genes reported association of a taste 1 receptor member 2 (*TAS1R2*)- rs11261087 variant with pancreatic cancer risk (Gentiluomo et al., 2019). Understanding baseline variations in chemosensory genes is likely to form an integral basis for precision nutrition for management of health and disease conditions.

Whole genome sequencing across different world populations provides a huge amount of baseline variation data. India houses a major fraction of global diversity. The IndiGen initiative of sequencing of 1,029 genomes has recently filled the gap in the variation data from Indian populations that are relatively under-represented in the global databases (Jain et al., 2021). We utilized this data to study the extent of variability in chemosensory genes that are likely to have evolved differently due to varied ethnicity, geo-climatic conditions, socio-cultural and culinary practices across India. We report patterns of genetic variations in 80 chemosensory genes across the representative IndiGenomes derived from diverse genetic clusters of the Indian reference populations (Indian Genome Variation Consortium, 2008). Our study shows differences from the global populations of the 1000 Genomes Project (The 1000 Genomes Project Consortium, 2015) and also highlights some key genes that could have evolved differently in Indian populations (Indian Genome Variation Consortium, 2008). This study provides important baseline genetic information on chemosensory genes that would be of great importance in precision nutrition studies.

## Materials and Methods

### Chemosensory Gene Set

80 genes associated with variations in taste perceptions and food preferences were manually curated from literature (Supplementary Table S1). The genes included have been associated with variations in 1) perception of five primary tastes: bitter, sweet, umami, salty, and sour 2) perceptions as well as preferences for food of different textures and odours and 3) variations in temperature thresholds and burning sensations in the mouth. Nearly 230 variants (SNPs) in these genes have earlier reported associations (Supplementary Table S2).

### Study Populations and Datasets

The study population included data of 1) 1,029 unrelated healthy Indian individuals from the IndiGen Genome Project (Jain et al., 2021) 2) 390 samples from 28 diverse populations of India included in the Indian Genome Variation (IGV) Consortium (Indian Genome Variation Consortium, 2008) 3) 2,504 samples from the 1000 Genomes project (The 1000 Genomes Project Consortium, 2015). The SAS populations of the 1,000 Genomes include four representative populations from South Asian ethnic groups and were also used to separately compare with the IndiGen genomes.

All the variants in the genes listed in Supplementary Table S1 and the variations residing in their flanking regions of 10 kb were retrieved from 1,029 unrelated healthy Indian individuals from the IndiGen Genome Project in variant call format (VCF, GRCh38/hg38). The IndiGen variants were prioritized after performing genotype level missingness tests. Variants with more than 10% missing genotypes were not included in the analysis. A total of 137,760 single nucleotide polymorphisms (SNPs) were finally used for analysis.

SNP genotypes were also retrieved for the same set of genes from the 1000 Genomes Project Phase 3 data (reference build GRCh37/hg19). 235,931 variants within 80 genes were obtained from 1,000 Genomes. 63,600 SNPs (46%) in the IndiGen dataset were common with 1,000 Genomes datasets. PLINKv1.09 (Purcell et al., 2007) and VCFTOOLS (Danecek et al., 2011) were used for variant filtering. We used the Liftover tool of UCSC (Hinrichs, 2006) for conversion of genomic coordinates of 1,000 Genomes data from hg19 to hg38. There were 2,898 variants that were shared between IndiGen and IGV. These were studied to estimate the frequency spectrum of chemosensory genes in diverse Indian ethnic groups. The frequency of the variants was calculated using VCFTOOLS and the SNPs were classified into three groups as common variants, rare variants and private variants based on their Minor Allele Frequency (MAF) cut-off of 5% and above, 1%–5%, and below 1% respectively. The number of variants in each group were compared between the 1,000 Genomes and IndiGen data to identify overlaps and differences between the two datasets as well as novel variants in the IndiGen data.

We further affirmed the novelty of unreported variants in IndiGen in two other publically available sources, the Simon Genome diversity Project (SGDP); *n* = 263 (37 of South Asian ancestry) and the Human Genome Diversity Project (HGDP); *n* = 929 (188 of South Asian ancestry).

### Variant Annotation

ANNOVAR (Wang et al., 2010) was used to annotate the variants using dbSNP v150 and RefGene annotations. The functional impact of exonic variations was estimated using CADD_phred scores annotated with the hg38_dbnsfp35a.txt database file. The variants that had a score above or equal to 20 were considered potentially deleterious. To assess differential enrichment of SNPs in genes and regulatory regions, the average percentage of SNPs in the genic and 10 kb flanking regions normalized to their lengths, were estimated across the different perception categories (bitter, sweet/fatty, salty/pungent, sour/astringent, and olfactory).

### Population Genetic Analysis

To estimate the population substructure within the IndiGen dataset, we carried out a conjoint analysis with the genome-wide common variants data that were shared between Indian Genome Variation (IGV), IndiGenomes and 1,000 Genomes populations. Combined variants (*n* = 100,800 SNPs) were used for principal component analysis (PCA) using PLINK v1.9 and the results were visualized using R-packages. To classify the IndiGen individuals based on their genetic ancestry, ADMIXTURE and PCA analyses were performed on a common dataset between IndiGen, 1,000 Genomes populations (YRI, CEU, FIN, CHB, and JPT) and samples from the Indian Genome Variation Consortium. The analysis was repeated with different sets of SNPs after applying HWE filters (*p-*value = 0.001) and LD r2 cut-off of 0.1, 0.2, 0.5. To assign the IndiGen samples to sub-populations of the IGV, PCA analysis followed by KNN calculations were performed. We further used the maximum likelihood methods implemented in ADMIXTURE software (Alexander et al., 2009) to estimate the individual-wise ancestry of IndiGen samples (Jain et al., 2021). The admixture analysis for each k was replicated 10 times and the k run which yielded the lowest CV error was selected to define the population structure. We also looked at signatures of selection in the chemosensory genes using different methods. The population differentiation (F_ST_) was estimated based on the Weir and Cockerhem method (1983) using VCFtools. Pairwise F_ST_ analysis was performed using the hierfstat of R package (Goudet, 2005). The ancestral allele information was obtained from the 1000 Genomes Project. Annotation of ancestral allele information was carried out using VCFTOOLS and the dataset for subsequent analysis was generated with PLINK. 48,824 sites with known ancestral alleles were used for derived allele frequency calculation.

### Divergence of Chemosensory Genes During Evolution

Protein sequences of chemosensory genes from 24 species that include primates and non-primates were retrieved using BLAST (Altschul et al., 1990). A phylogenetic analysis was performed to study the divergence patterns. A similar number of a random set of non-chemosensory genes were also included.

## Results

### Pattern of Variations in Chemosensory Genes Among the Indian Genomes

**FIGURE 1:**
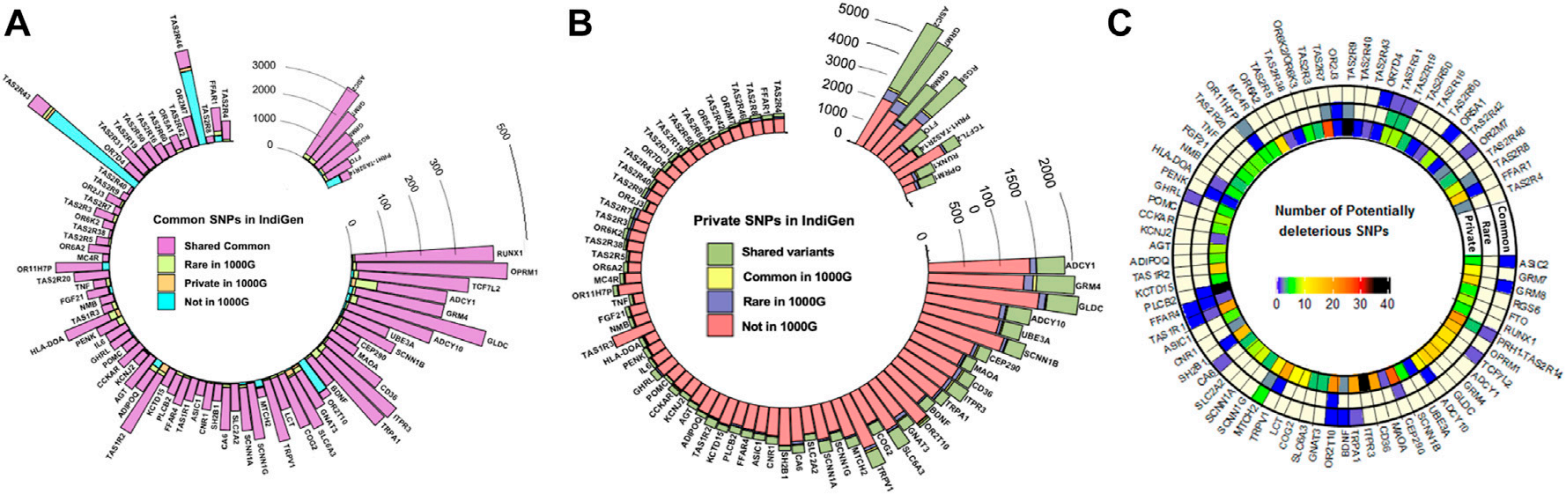
Comparison of the frequency distribution of variants across the genes in IndiGen and the 1000 Genomes Project (1000G) **(A)** Number of SNPs with a common frequency in IndiGen **(B)** Number of private SNPs in IndiGen and their frequencies in other populations of the 1000G—The same frequency category as in IndiGen or higher/lower frequencies than in IndiGen **(C)** The numbers of potentially deleterious variants across the genes. The genes are ordered by size from largest to smallest (clockwise).

A total of 137,760 SNPs in the 80 chemosensory genes including 10 kb upstream and downstream regions were retrieved from 1,000 IndiGen genomes. In these genes, the 1,000 Genomes Phase 3 data (*n* = 2015) and SAS population (*n* = 489) harbored a corresponding set of 203,560 and 75,555 SNPs respectively. There were ∼33,000 insertion and deletion polymorphisms comprising 18% of the total variants. These were not included in the present analysis. 68% of the variants were intronic and 2% were exonic as anticipated. Nearly 75% of variants were C to T transitions. The details of annotation and distribution of variants in chemosensory genes in IndiGen data have been depicted in Supplementary Figures S1A–D. Surprisingly, out of the 2,528 exonic variants, the fraction of nonsynonymous (NS) variants was higher than synonymous. A smaller fraction of the variants in the exonic regions were also frameshift causing insertions and deletions. There were nearly 65 nonsense mutants in the NS class and few splice site variants. Ten top mutated genes had more than 35 variants and included *CEP290*, *ITPR3*, *TAS1R2*, *TRPV1*, and *CD36* (Supplementary Figure S2). There did not seem to be any correlation with the number of potentially deleterious exonic variants to the size of the genes. A large fraction of the variants was private and the density of variation was observed to differ across genes. *MTCH1* harbours a higher number of potentially deleterious common variants, while *CEP290*, *ITPR3*, *PLCB2*, *TAS2R7*, and *TAS2R9* have higher overall numbers (Figure 1C). The average percentage of SNPs in the genic and 10 kb flanking regions, normalized for the length across the different perception categories (bitter, sweet/fatty, salty/pungent, sour/astringent, and olfactory) were also calculated. It was observed that the genes associated with olfaction harbored more SNPs (1.61%) followed by salt taste and pungency associated genes (*SCNN1A*, *SCNN1B*, *SCNN1G*, *TRPV1*) (1.17%).

### Chemosensory Gene Variants in IndiGenomes vs. 1,000 Genomes Populations: Similarities and Uniqueness

Nearly 70% (17,774) of the common variants all-together in the study are shared between IndiGen and 1,000 genomes (Figure 1 and Supplementary Figure S3A), which is 13% of all the variants in IndiGen. Interestingly, 8.2% of the common variants are also unique to IndiGenomes and not present in any of the 1,000 Genomes populations. An additional 3% IndiGen variants overlap with SAS. However, 10% of the common variants shared between SAS and non-SAS populations of the 1,000 Genomes are not observed in IndiGen. The overlap of rare variants between the three cohorts is 9.6%. With respect to rare variants, the overlap is much more between SAS and non-SAS populations of 1,000 genomes. Only 3% of the private variants are shared across IndiGen and SAS and non-SAS of 1,000 genomes datasets. IndiGen data has a significantly higher proportion of unique private variants (27%) (Figure 1). In all the three sets, the extent of overlap of private variants in IndiGene with SAS is higher (9%) (Supplementary Figure S3A).

Amongst all the genes and the considered flanking regions, the bitter taste receptors in the IndiGen data have a higher number of common and rare variants compared to SAS and 1000G. The olfactory receptor gene *OR2T10,* has a higher number of SNPs in IndiGen in all three categories (Supplementary Figure S3A). The bitter-associated genes *PRH1-TAS2R14* (which encompasses *TAS2R43* and *TAS2R46*), *OR2T10*, *OR11HP* (olfaction)*, MTCH2*, *FFAR1*, *ADIPOQ* (sweet and fatty foods) have a higher number of novel common variants. Most of the genes are found to have higher proportions of private variants in IndiGen not represented in the 1000 Genomes Project, while some genes like *FTO*, *RGS6*, *OPRM1* (sweet and fatty foods)*, GRM7*, *GRM8*, *ASIC2*, *RUNX1* (sour/marmite) have more private variants shared between the two datasets (Figures 1A,B).

We also analyzed the frequency distribution of 231 variants that have been previously associated with taste perceptions. Out of 231 variants, 195 variants were observed in the IndiGen dataset that also includes 3 monomorphic variants (Supplementary Table S2). Seven variants had a frequency less than 1% and the rest 185 variants had a frequency greater than 1% in the IndiGen dataset. Amongst these, 61 variants were exonic variants with 49 nonsynonymous, 11 synonymous and a stop-gain variant. Out of the 49 nonsynonymous variants, 8 were not observed in IndiGen.

To check if the variants unique to IndiGen compared to the 1,000 Genomes dataset are novel and not reported elsewhere, we also compared the variants with HGDP and SGDP. 46% of the IndiGen variants were found to be novel (Supplementary Figure S3C), most of them being private (<1% MAF).

### Mapping Variations in Chemosensory Perception to Genetic Ancestry: Relatedness and Admixture Analysis of IndiGenomes

**FIGURE 2:**
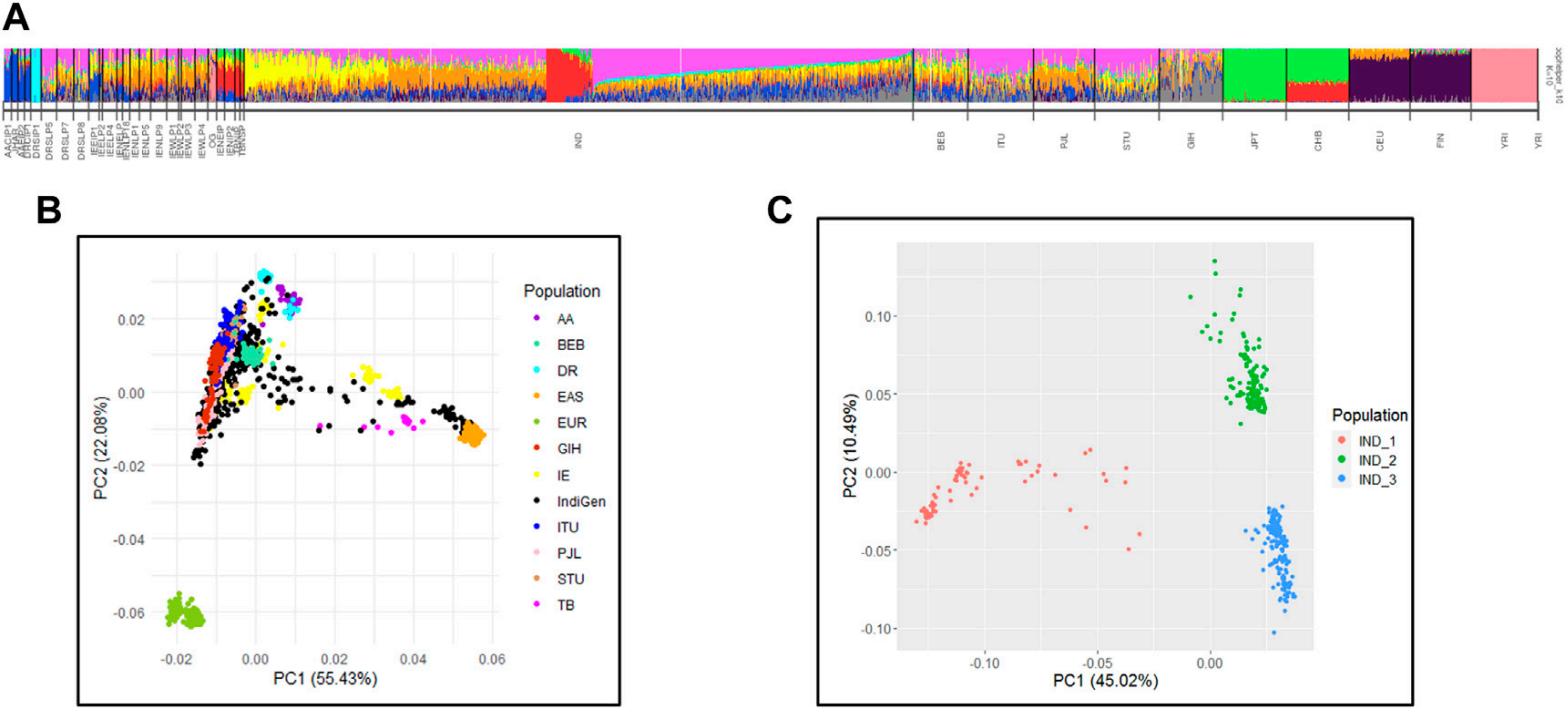
Population genetic structure interpretation of IndiGen with IGV and 1000G datasets by **(A)** ADMIXTURE analysis and **(B)** PCA **(C)** PCA plot showing three IndiGen groups of pure ancestral components.

In previous studies of the Indian Genome Variation Consortium (IGVC) we have reported that the Indian population maps to five major genetic clusters (Indian Genome Variation Consortium, 2008). We performed conjoint analysis of the 1,000 Genomes dataset with IGV and IndiGen to map ancestry and admixture proportions in the IndiGenomes. In the PCA analysis, PC1 separated African versus non-African populations while PC2 separated Europeans with East-Asian population (Supplementary Figure S4). None of the IndiGen individuals show closeness to African (YRI) or admixed African Indian (OG) populations. In the subsequent PCA analysis, a subset of IndiGen individuals show their closeness to East Asians and Tibeto-Burman populations of IGV in PC1, while second group of IndiGen Individuals are closer to European of 1,000 Genomes and Indo-Europeans large populations of IGV in PC2 (Figure 2B). The third set of IndiGen samples are closer to IGV populations of Dravidian large population linguistic groups while the rest of the IndiGen individuals fall within the Indo-European and Dravidian linguistic cline. Individual-wise ancestry of IndiGen samples were further assessed using maximum likelihood-based methods implemented in ADMIXTURE. The lowest cross validation (CV) error was observed at K = 10 and used for structure interpretation (Supplementary Figure S5). A similar ancestry component was observed in admixture analysis where three predominant groups namely, Tibeto-Burman like (IND_1 group), Indo-European like (IND_2 group), and Dravidian like (IND_3 group) were classified (Figure 2C). The individual-wise ancestry estimates of these three predominant individuals are depicted in Supplementary Figure S6. The three predominant groups were used for further population comparisons analysis of chemosensory gene variants. Pairwise F_ST_ analysis shows that IndiGen individuals have maximal closeness with SAS populations of the 1,000 Genomes.

Derived allele frequency (DAF) of the variants within chemosensory genes were calculated which were further used to calculate the absolute delta DAF between pairwise populations. Variants were prioritized based on signatures of positive selection with both F_ST_ value ≥0.3 and delta DAF ≥0.5 between pairwise populations. Pairwise population comparisons provide 1,184 variants within chemosensory genes that show high F_ST_ and high derived allele frequency in IndiGen compared to other world populations (Supplementary Table S3). Genes with variants showing high values include *ADCY10*, *TRPV1*, *RUNX1*, *GRM7*, *TCF7L2*, *OR7D4*, *ASIC2*, and *CEP290* (Supplementary Table S3)*.* Many variants within the same genes and flanking region are in linkage disequilibrium with each other. 17 variants from previously reported association on taste perception showed high F_ST_ and derived allele frequency in IndiGen populations (Table 1). Nine functionally important nonsynonymous variants within chemosensory genes that have not been reported previously show high differentiation (Table 1). Further comparison of population differentiation of variants within IndiGen sub-populations reveals 187 variants with F_ST_ value ≥0.3 (Supplementary Table S4).

**TABLE 1:** High F_ST_ variants differentiating world populations either earlier reported to be associated with taste perception or nonsynonymous functional variations.

SNP ID	Location	Gene	Reported associations with the SNP	Taste perception associated with the SNP/gene
rs11064153	intergenic	Near *SCNN1A*	Yang et al., 2014 (33)	Salt taste
rs10256873	Intronic	*GRM8*	Roos et al., 2017 (34)	Marmite taste
rs11760281	Intronic	*GNAT3*	Farook et al., 2012	Sucrose intake
rs150908	Intronic	*TRPV1*	Chamoun et al., 2018	Salt taste
rs1524600	Intronic	*GNAT3*	Knaapila et al., 2012	Sweet taste
rs224549	Intronic	*TRPV1*	Allen et al., 2014 ^a^	Pungent and bitter taste
rs224550	Intronic	*TRPV1*	Allen et al., 2014 ^a^	Pungent and bitter taste
rs224551	Intronic	*TRPV1*	Allen et al., 2014 ^a^	Pungent and bitter taste
rs2277675	Intronic	*TRPV1*	Pirastu et al., 2012	Salt taste
rs3211913	Intronic	*CD36*	(Love‐Gregory et al., 2008)	Fat taste perception
rs3818521	Intronic	*ITPR3*	Pirastu et al., 2012	Lamb meat and sheep cheese liking
rs4908563	Intronic	*TAS1R1*	Chamoun et al., 2018	Sweet/Umami Taste
rs769148	Intronic	*RGS6*	Sibbel et al., 2011	Higher intake of fat/oil/sweets
rs236514	UTR3	*KCNJ2*	Chamoun et al., 2018	Sour taste
rs2274333	nonsynonymous	*CA6*	Melis et al., 2013	PROP Sensitivity (Bitter)
rs1799971	nonsynonymous	*OPRM1*	Deb et al., 2010	Alcohol taste
rs2233998	nonsynonymous	*TAS2R4*	Choi et al., 2017	Bitter taste
rs936212	nonsynonymous	*PLCB2*	None reported	Sweet
rs10845281	nonsynonymous	*TAS2R20*	None reported	Bitter taste
rs10845279	nonsynonymous	*TAS2R20*	None reported	Bitter taste
rs2234002	nonsynonymous	*TAS2R4*	None reported	Bitter taste
rs35969491	nonsynonymous	*TAS2R42*	None reported	Bitter taste
rs1376251	nonsynonymous	*TAS2R50*	None reported	Bitter taste
rs224534	nonsynonymous	*TRPV1*	None reported	Salt taste
rs9262	nonsynonymous SNV	*C12orf29* (Upstream of *CEP290*)	None reported	Marmite

a In LD with rs224547 in the SAS population which is reported to be significantly associated with burning/stinging and bitterness.

**FIGURE 3:**
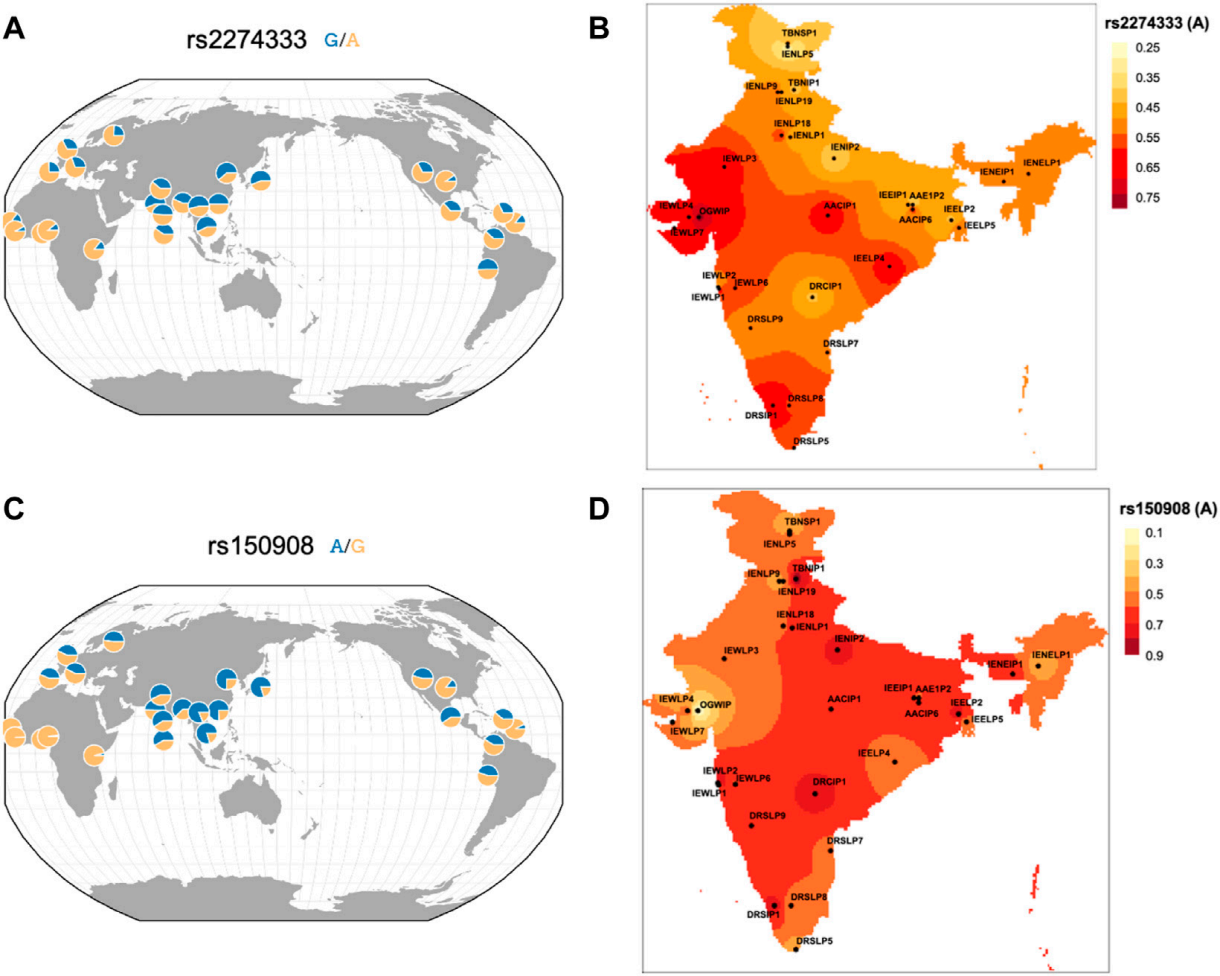
Spatial plot of frequency distribution of high differentiating variants **(A)** rs2274333 of CA6 gene in worldwide geographical population and **(B)** in IGVC populations of India and **(C)** rs150908 of TRPV1 gene in worldwide geographical population and **(D)** in IGVC populations.

165 variants with high F_ST_ and delta DAF variants also F_ST_ in the Indian Genome Variation Consortium (IGV) data were also studied (Supplementary Table S4). This allows assessment across a larger fraction of population as IGV has a larger representation of contrasting and diverse ethnic populations sampled across India. Out of 165 variants, 7 have been reported previously which also includes rs2274333 and rs150908 (Table 1 and Figure 3). As depicted in Figure 3 the frequencies of derived alleles of corresponding variants display striking differences among world populations and different ethnic populations from India. These variants could play an important role in taste perception and dietary preferences among the human population and may be biologically important.

### Divergence of Bitter Taste Receptor Genes Among Chemosensory Genes

**FIGURE 4:**
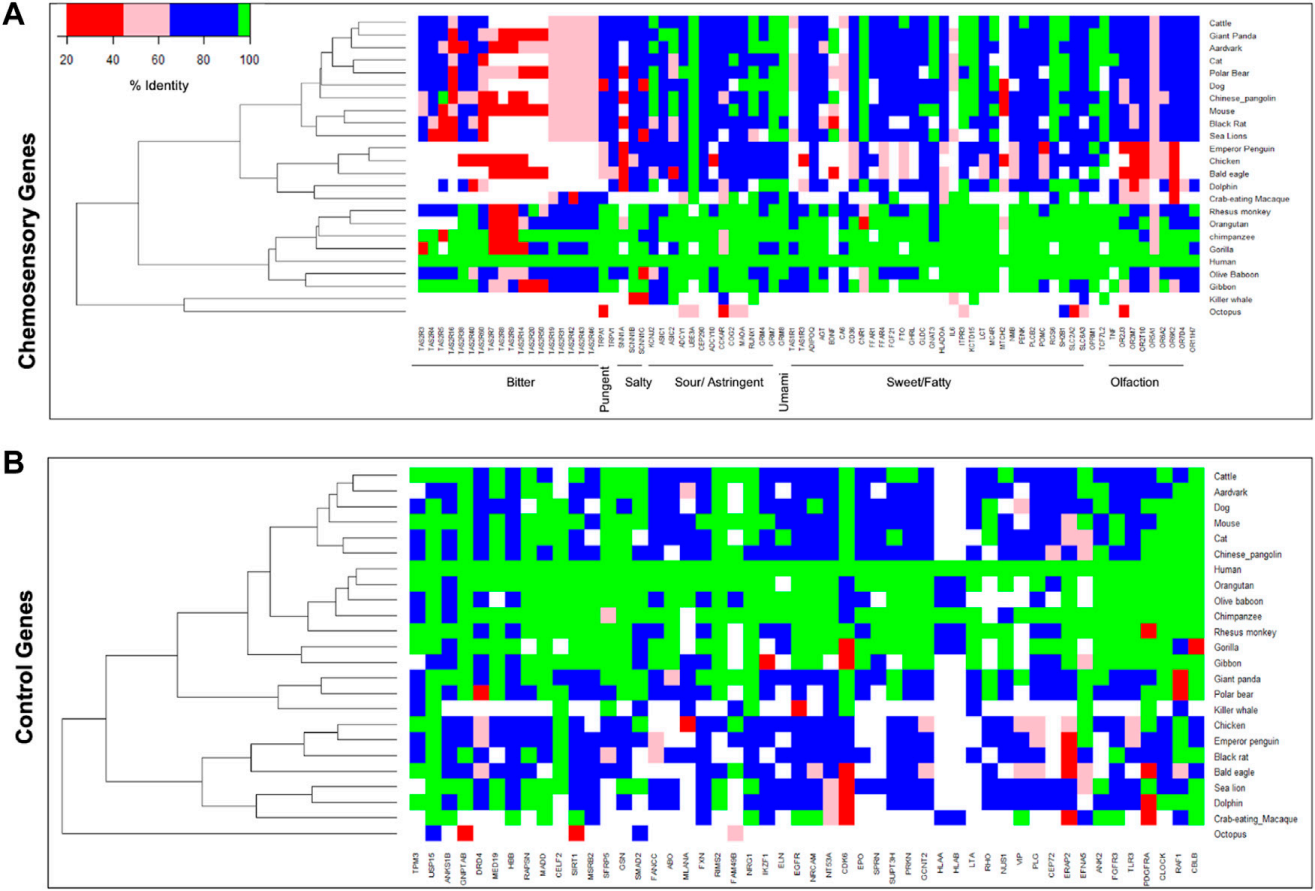
Heatmaps represent the protein sequence diversity of **(A)** chemosensory perception-associated genes and **(B)** the control set of genes across different species. The color represents lower (red) to higher (green) protein sequences conservation.

To understand the divergence of chemosensory genes during organisms’ evolution, the protein sequences of humans with 24 other organisms that include primate and non-primate species were compared. The chemosensory genes responsible for bitter taste show more divergence compared to the other groups, followed by olfactory receptor genes (Figure 4). However, such differences are not observed for a random set of non-chemosensory and other categories of chemosensory genes. This suggests that genes responsible for bitter taste perception diversify more according to their food preferences in the environment.

## Discussion and Conclusion

Here, we studied patterns of genetic variability in 80 genes previously known to be associated with chemosensory perceptions in the recently available whole-genome sequencing data of 1,029 healthy Indian individuals (IndiGen) and compared these with other ethnic world populations. We observe a total of 137,760 SNPs within chemosensory genes in the IndiGen dataset including private variants. 9% of the common SNPs and 27% private variants in IndiGen seem to be Indian population specific as they are not represented in 1,000 Genomes further substantiating the diversity and uniqueness of IndiGen data even for chemosensory genes (Jain et al., 2021). Further, the bitter taste receptor genes display higher variation compared to other taste receptor gene sets. The protein sequence diversity estimates between human and other species also reveal higher divergence of bitter taste receptor genes compared to other chemosensory genes and control gene sets which reflects dietary habits of different species as well as provide potential variants that are involved in sensing of bitter taste to avoid ingestion of harmful food (Dong et al., 2009).

Conjoint population genetic analysis of IndiGen individuals with those from Indian Genome Variation Consortium and 1,000 Genomes identified sets of individuals that show proximity to three predominant ethnic groups namely, Tibeto-Burman group (IND_1), Indo-European large group (IND_2), and Dravidian large group (IND_3).

We speculated that differences in genetic variation and diversity of chemosensory genes between IndiGen data and other ethnic populations could also reflect differences in selective pressure. We thus looked at the extent of differentiation through pairwise F_ST_ and derived allele frequency statistics. Based on higher F_ST_ value and higher delta DAF between pairwise population comparison we identified 1,181 variants within chemosensory genes that display differences in derived allele frequency between IndiGen populations and representative European (CEU), African (YRI) and East Asian (CHB) populations. The top genes were *ADCY10*, *TRPV1*, *RUNX1*, *GRM7*, *TCF7L2*, *OR7D4*. These could be potential targets of positive selection.

The baseline variability information in chemosensory genes could have utility in nutrigenomic and precision medicine studies especially the ones that show high F_ST_ values (Table 1). For example, a variant rs3818521 *ITPR3* has been studied for its association with lamb-meat and cheese-liking (umami). Its minor allele (C) reported to be associated with lamb meat liking (Pirastu et al., 2012) has lower frequency in the Indian population and highest in African. Another interesting SNP was rs769148 in *RGS6* wherein the carriers of C allele are prone to obesity development due to higher intake of fat/oil/sweets that are palatable energy-dense foods (Sibbel et al., 2011). The frequency of derived allele T was highest (81%) in Ind_1 of IndiGen set who are related to the Tibeto-Burman lineage. This is also highest in its most proximal East Asian (CHB; 93%) population from 1,000 Genomes. The T allele is associated with lower preferences for fat/oily food thereby contributing to reduced risk for obesity in these populations. A recent study has reported a greater prevalence of the G allele of rs1799971 (*OPRM1*) in both alcoholic and opioid addicts in India compared with those in the normal population (Deb et al., 2010) that also corroborates with similar findings from a study in Sweden (Bart et al., 2005). This allele has also been reported to be associated with a higher preference for sweet and fatty foods (Davis et al., 2011). The frequency spectrum of this variant displays differences amongst diverse Indian Populations in the IGVC (Supplementary Table S5). When looking at the variants with reported association in IGV populations, some of them seem to be more striking. Among them, rs2274333 of the *CA6* (Carbonic Anhydrase 6) gene is associated with PROP sensitivity where the AA genotype (homozygous ancestral allele associated with a fully-functional gustin protein) and the GG genotypes are more frequent in supertasters and nontasters respectively. This suggests that variation in gustin may be associated with differences in papillae densities and oral chemosensory abilities in individuals sensing PROP (Melis et al., 2013). The frequency of derived allele was higher in Asian populations compared to Europeans and Africans (Figure 3A) however, the frequency of derived allele was much higher in Indo-Europeans of North and North-Eastern populations and lower in Indo-Europeans of Western India. Variation in frequency spectrum suggests that populations from western part could have more supertasters compared to the north. Even within the Dravidian population, differences are observed. This polymorphism could also affect the intake of coffee and white cabbage (Mikołajczyk-Stecyna et al., 2017). The A allele of rs150908 (*TRPV1*) is associated with a higher salt sensitivity and hence a lower preference for salty foods (Chamoun et al., 2018). Another *TRPV1* variant rs2277675 is likely to be associated with sweet perception as it’s minor allele (C) is associated with less liking and preference for beet (Pirastu et al., 2012). It would be interesting to see whether some of these variations could contribute to altered perceptions between individuals and populations. Likewise, the C allele of rs4790522 (*TRPV1*) is known to be associated with high salt preference and a risk for cardiovascular diseases (Precone et al., 2019). Generally with the increase in altitude, taste perception for salt decreases. The frequency of this allele was higher, mostly in the North-Eastern parts of India (high altitude) (Supplementary Figure S7). We anticipate that conducting chemosensory/taste perception tests on a large scale and correlation with the underlying genotypes would shed light on the genetic basis of chemosensory perceptions and food preferences and would be useful in the field of precision nutrition.

As taste, flavours and texture forms the basis of determination of food selection and dietary habits, inter-individual variations in chemosensory perception can prove to be highly useful in the characterization of differences in nutrient intake. Since this is also directly linked to the health and general well-being of an individual, evaluation of these polymorphisms in taste receptor genes can assist in understanding the association between food selection and the occurrence of numerous metabolic disorders. Moreover, a comprehensive knowledge of genetic variations in taste receptor genes can aid the development of personalized nutrition models that can help design dietary recommendations for preempting disorders due to dietary habits and enhance the general quality of life of individuals. A catalogue of curated variants could thus be enormously helpful in discerning differences in food preferences both within a population and between different global populations.

## Web Resources

IndiGen database: http://clingen.igib.res.in/indigen/

1,000 Genomes database: ftp://ftp-trace.ncbi.nih.gov/1000genomes/ftp/release/20130502/

1,000 Genomes ancestral allele information: ftp://ftp.1000genomes.ebi.ac.uk

IGV database: http://igvdb.res.in/

PLINK software: http://zzz.bwh.harvard.edu/plink/ www.cog-genomics.org/plink/1.9/

VCFTOOLS: http://vcftools.sourceforge.net/

UCSC Genome Browser: https://genome.ucsc.edu/

ANNOVAR: https://annovar.openbioinformatics.org/

GGV: http://popgen.uchicago.edu/ggv/

## Data Availability

The SNP information from the IndiGen project (Jain et. Al., 2020) are available at https://clingen.igib.res.in/indigen/ and the genotype data can be made available upon request. The codes used for the analyses can be accessed at https://github.com/Prakrithi-P/IndiGen_Chemosensory-Landscape and the results from the current study can be found in the Supplementary Material.
