# Supplementary material for: Landscape of Variability in Chemosensory Genes Associated With Dietary Preferences in Indian Population: Analysis of 1029 Indian Genomes
Source: Front Genet. 2022 Jul 12;13:878134. doi: 10.3389/fgene.2022.878134 (PMC9315315; doi:10.3389/fgene.2022.878134)
Supplement: Supplementary file 3 [file Table1.docx]

### Supplementary Material

#### Supplemental Table 1: List of genes used in the study, with reported variants associated with chemosensory perceptions and food preferences

| **Perception** | **Gene name** |
| --- | --- |
| Bitter | *TAS2R3, TASR4, TAS2R5, TAS2R7, TAS2R8, TAS2R9, TAS2R14, PRHI-TAS2R14,*  *TAS2R16, TAS2R19, TAS2R20, TAS2R31, TAS2R38, TAS2R42, TAS2R43, TAS2R46,*  *TAS2R50, TAS2R60, TRPA1, GNAT3, CA6, TRPV1* [(Hinrichs et al., 2006; Hayes et al., 2011; Knaapila et al., 2012; Keller et al., 2013; Melis et al., 2013; Allen et al., 2014; Choi and Chan, 2015; Barontini, 2017; Choi et al., 2017; Mikołajczyk-Stecyna et al., 2017; Barragán et al., 2018; Bartáková et al., 2018; Chamoun et al., 2018b, 2018a; Hwang et al., 2018; Eriksson et al., 2019; Precone et al.)](https://www.zotero.org/google-docs/?VsjgAb). |
| Sweet | *TAS1R2, TAS1R3,ADIPOQ, NMB, FGF21, GHRL, FTO, LCT, SLC6A3,SLC2A2, TCF7L2,OPRM1, RGS6, POMC,PENK, TRPV1, BDNF,ITPR3* and *PLCB2* (signal transduction) [(Bienertova-Vasku et al., 2009; Fushan et al., 2009; Landgren et al., 2011; Dias et al., 2015; Fontana et al., 2015; Choi et al., 2017; Davis et al., 2017; Han et al., 2017; Bartáková et al., 2018; Chamoun et al., 2018b, 2018a; Eriksson et al., 2019; Davis; Melo et al.; Precone et al.)](https://www.zotero.org/google-docs/?mpOnAT) |
| Umami | *TAS1R1, TAS1R3, GRM4, FTO* [(Chen et al., 2009; Chamoun et al., 2018b, 2018a; Grippi et al., 2019; Melo et al.; Precone et al.)](https://www.zotero.org/google-docs/?x9sWk1). |
| Fatty/Creamy/Energy dense Food | *CD36,FFAR1,FFAR4,AGT, MC4R, SH2B1, KCTD15, FTO, IL-6,RGS6, TNF-α, POMC, GNAT3, TRPV1,OPRM1,CNR1* [(Ma et al., 2004; Bokor et al., 2010; Caruso et al., 2012; Keller et al., 2012; Gautam et al., 2013, 2015; Jayewardene et al., 2014, 2016; Lee et al., 2015; Mrizak et al., 2015; Kirac et al., 2016; Park et al., 2016; Davis et al., 2017; Bartáková et al., 2018; Chamoun et al., 2018b, 2018a; Plesník et al., 2018; Mulianah Daya et al., 2019; Bauer et al.; Davis; Love-Gregory et al.; Precone et al.)](https://www.zotero.org/google-docs/?UEEStg). |
| Salty | *SCNN1A, SCNN1B, SCNN1G, TRPV1* [(Dias et al., 2013; Yang et al., 2014; Chamoun et al., 2018b, 2018a)](https://www.zotero.org/google-docs/?yz8X1I). |
| Sour | *ASIC1, KCNJ2* [*(Chamoun et al., 2018b, 2018a; Eriksson et al., 2019)*](https://www.zotero.org/google-docs/?moQm39) |
| Pungent/ Burning sensations | *TRPV1* [*(Allen et al., 2014)*](https://www.zotero.org/google-docs/?eDl3eg)*.* |
| Olfaction | *OR5A1, OR6A2, OR11H7, OR2J3, OR7D4, OR6K2,OR6K3, OR2M7* [(Menashe et al., 2003; Lunde et al., 2012; Jaeger et al., 2013; Ignatieva et al., 2014; Precone et al.)](https://www.zotero.org/google-docs/?DaSwW2), *HLA-DOA* [(Pirastu et al., 2015)](https://www.zotero.org/google-docs/?mx3i64)*.* |
| Marmite | *CEP290, RUNX1, MAOA, UBE3A, GLDC, COG2, OR2T10,ADCY1,ADCY10,CCKAR,ASIC2,GRM7,GRM8* [(Roos et al., 2017)](https://www.zotero.org/google-docs/?9vIfon). |

**Supplemental References**

[Allen, A. L., McGeary, J. E., and Hayes, J. E. (2014). Polymorphisms in TRPV1 and TAS2Rs Associate with Sensations from Sampled Ethanol. *Alcohol. Clin. Exp. Res.* 38, 2550–2560. doi:10.1111/acer.12527.](https://www.zotero.org/google-docs/?iJx1rw)

[Barontini, J. (2017). Association between polymorphisms of TAS2R16 and susceptibility to colorectal cancer. 7.](https://www.zotero.org/google-docs/?iJx1rw)

[Barragán, R., Coltell, O., Portolés, O., Asensio, E., Sorlí, J., Ortega-Azorín, C., et al. (2018). Bitter, Sweet, Salty, Sour and Umami Taste Perception Decreases with Age: Sex-Specific Analysis, Modulation by Genetic Variants and Taste-Preference Associations in 18 to 80 Year-Old Subjects. *Nutrients* 10, 1539. doi:10.3390/nu10101539.](https://www.zotero.org/google-docs/?iJx1rw)

[Bartáková, V., Kuricová, K., Zlámal, F., Bělobrádková, J., and Kaňková, K. (2018). Differences in food intake and genetic variability in taste receptors between Czech pregnant women with and without gestational diabetes mellitus. *Eur. J. Nutr.* 57, 513–521. doi:10.1007/s00394-016-1334-6.](https://www.zotero.org/google-docs/?iJx1rw)

[Bauer, F., Elbers, C. C., Adan, R. A., Loos, R. J., Onland-Moret, N. C., Grobbee, D. E., et al. Obesity genes identiﬁed in genome-wide association studies are associated with adiposity measures and potentially with nutrient-speciﬁc food preference1–3. 9.](https://www.zotero.org/google-docs/?iJx1rw)

[Bienertova-Vasku, J., Bienert, P., Tomandl, J., Forejt, M., and Vasku, A. (2009). Relation between adiponectin 45 T/G polymorphism and dietary composition in the Czech population. *Diabetes Res. Clin. Pract.* 84, 329–331. doi:10.1016/j.diabres.2009.02.023.](https://www.zotero.org/google-docs/?iJx1rw)

[Bokor, S., Legry, V., Meirhaeghe, A., Ruiz, J. R., Mauro, B., Widhalm, K., et al. (2010). Single-nucleotide Polymorphism of CD36 Locus and Obesity in European Adolescents. *Obesity* 18, 1398–1403. doi:10.1038/oby.2009.412.](https://www.zotero.org/google-docs/?iJx1rw)

[Caruso, M. G., Gazzerro, P., Notarnicola, M., Cisternino, A. M., Guerra, V., Misciagna, G., et al. (2012). Cannabinoid Type 1 Receptor Gene Polymorphism and Macronutrient Intake. *J. Nutr. Nutr.* 5, 305–313. doi:10.1159/000343563.](https://www.zotero.org/google-docs/?iJx1rw)

[Chamoun, E., Carroll, N., Duizer, L., Qi, W., Feng, Z., Darlington, G., et al. (2018a). The Relationship between Single Nucleotide Polymorphisms in Taste Receptor Genes, Taste Function and Dietary Intake in Preschool-Aged Children and Adults in the Guelph Family Health Study. *Nutrients* 10, 990. doi:10.3390/nu10080990.](https://www.zotero.org/google-docs/?iJx1rw)

[Chamoun, E., Mutch, D. M., Allen-Vercoe, E., Buchholz, A. C., Duncan, A. M., Spriet, L. L., et al. (2018b). A review of the associations between single nucleotide polymorphisms in taste receptors, eating behaviors, and health. *Crit. Rev. Food Sci. Nutr.* 58, 194–207. doi:10.1080/10408398.2016.1152229.](https://www.zotero.org/google-docs/?iJx1rw)

[Chen, Q.-Y., Alarcon, S., Tharp, A., Ahmed, O. M., Estrella, N. L., Greene, T. A., et al. (2009). Perceptual variation in umami taste and polymorphisms in TAS1R taste receptor genes. *Am. J. Clin. Nutr.* 90, 770S-779S. doi:10.3945/ajcn.2009.27462N.](https://www.zotero.org/google-docs/?iJx1rw)

[Choi, J.-H., Lee, J., Yang, S., and Kim, J. (2017). Genetic variations in taste perception modify alcohol drinking behavior in Koreans. *Appetite* 113, 178–186. doi:10.1016/j.appet.2017.02.022.](https://www.zotero.org/google-docs/?iJx1rw)

[Choi, S. E., and Chan, J. (2015). Relationship of 6-n-Propylthiouracil Taste Intensity and Chili Pepper Use with Body Mass Index, Energy Intake, and Fat Intake within an Ethnically Diverse Population. *J. Acad. Nutr. Diet.* 115, 389–396. doi:10.1016/j.jand.2014.09.001.](https://www.zotero.org/google-docs/?iJx1rw)

[Davis, C. Opiates, overeating and obesity: a psychogenetic analysis. *Int. J. Obes.*, 8.](https://www.zotero.org/google-docs/?iJx1rw)

[Davis, C., Patte, K., Zai, C., and Kennedy, J. L. (2017). Polymorphisms of the oxytocin receptor gene and overeating: the intermediary role of endophenotypic risk factors. *Nutr. Diabetes* 7, e279–e279. doi:10.1038/nutd.2017.24.](https://www.zotero.org/google-docs/?iJx1rw)

[Dias, A. G., Eny, K. M., Cockburn, M., Chiu, W., Nielsen, D. E., Duizer, L., et al. (2015). Variation in the TAS1R2 Gene, Sweet Taste Perception and Intake of Sugars. *Lifestyle Genomics* 8, 81–90. doi:10.1159/000430886.](https://www.zotero.org/google-docs/?iJx1rw)

[Dias, A. G., Rousseau, D., Duizer, L., Cockburn, M., Chiu, W., Nielsen, D., et al. (2013). Genetic Variation in Putative Salt Taste Receptors and Salt Taste Perception in Humans. *Chem. Senses* 38, 137–145. doi:10.1093/chemse/bjs090.](https://www.zotero.org/google-docs/?iJx1rw)

[Eriksson, L., Esberg, A., Haworth, S., Holgerson, P. L., and Johansson, I. (2019). Allelic Variation in Taste Genes Is Associated with Taste and Diet Preferences and Dental Caries. *Nutrients* 11, 1491. doi:10.3390/nu11071491.](https://www.zotero.org/google-docs/?iJx1rw)

[Fontana, C., Vitolo, M. R., Campagnolo, P. D. B., Mattevi, V. S., Genro, J. P., and Almeida, S. (2015). DRD4 and SLC6A3 gene polymorphisms are associated with food intake and nutritional status in children in early stages of development. *J. Nutr. Biochem.* 26, 1607–1612. doi:10.1016/j.jnutbio.2015.07.030.](https://www.zotero.org/google-docs/?iJx1rw)

[Fushan, A. A., Simons, C. T., Slack, J. P., Manichaikul, A., and Drayna, D. (2009). Allelic Polymorphism within the TAS1R3 Promoter Is Associated with Human Taste Sensitivity to Sucrose. *Curr. Biol.* 19, 1288–1293. doi:10.1016/j.cub.2009.06.015.](https://www.zotero.org/google-docs/?iJx1rw)

[Gautam, S., Agrawal, C. G., and Banerjee, M. (2015). CD36 Gene Variants in Early Prediction of Type 2 Diabetes Mellitus. *Genet. Test. Mol. Biomark.* 19, 144–149. doi:10.1089/gtmb.2014.0265.](https://www.zotero.org/google-docs/?iJx1rw)

[Gautam, S., Pirabu, L., Agrawal, C. G., and Banerjee, M. (2013). CD36 Gene Variants and Their Association with Type 2 Diabetes in an Indian Population. *Diabetes Technol. Ther.* 15, 680–687. doi:10.1089/dia.2012.0326.](https://www.zotero.org/google-docs/?iJx1rw)

[Grippi, C., Ahrens, W., Buchecker, K., Chadjigeorgiou, C., De Henauw, S., Koni, A. C., et al. (2019). Association between variants of neuromedin U gene and taste thresholds and food preferences in European children: Results from the IDEFICS study. *Appetite* 142, 104376. doi:10.1016/j.appet.2019.104376.](https://www.zotero.org/google-docs/?iJx1rw)

[Han, P., Keast, R. S. J., and Roura, E. (2017). Salivary leptin and *TAS1R2/TAS1R3* polymorphisms are related to sweet taste sensitivity and carbohydrate intake from a buffet meal in healthy young adults. *Br. J. Nutr.* 118, 763–770. doi:10.1017/S0007114517002872.](https://www.zotero.org/google-docs/?iJx1rw)

[Hayes, J. E., Wallace, M. R., Knopik, V. S., Herbstman, D. M., Bartoshuk, L. M., and Duffy, V. B. (2011). Allelic Variation in TAS2R Bitter Receptor Genes Associates with Variation in Sensations from and Ingestive Behaviors toward Common Bitter Beverages in Adults. *Chem. Senses* 36, 311–319. doi:10.1093/chemse/bjq132.](https://www.zotero.org/google-docs/?iJx1rw)

[Hinrichs, A. L., Wang, J. C., Bufe, B., Kwon, J. M., Budde, J., Allen, R., et al. (2006). Functional Variant in a Bitter-Taste Receptor (hTAS2R16) Influences Risk of Alcohol Dependence. *Am. J. Hum. Genet.* 78, 103–111. doi:10.1086/499253.](https://www.zotero.org/google-docs/?iJx1rw)

[Hwang, L.-D., Gharahkhani, P., Breslin, P. A. S., Gordon, S. D., Zhu, G., Martin, N. G., et al. (2018). Bivariate genome-wide association analysis strengthens the role of bitter receptor clusters on chromosomes 7 and 12 in human bitter taste. *BMC Genomics* 19, 678. doi:10.1186/s12864-018-5058-2.](https://www.zotero.org/google-docs/?iJx1rw)

[Ignatieva, E. V., Levitsky, V. G., Yudin, N. S., Moshkin, M. P., and Kolchanov, N. A. (2014). Genetic basis of olfactory cognition: extremely high level of DNA sequence polymorphism in promoter regions of the human olfactory receptor genes revealed using the 1000 Genomes Project dataset. *Front. Psychol.* 5. doi:10.3389/fpsyg.2014.00247.](https://www.zotero.org/google-docs/?iJx1rw)

[Jaeger, S. R., McRae, J. F., Bava, C. M., Beresford, M. K., Hunter, D., Jia, Y., et al. (2013). A Mendelian Trait for Olfactory Sensitivity Affects Odor Experience and Food Selection. *Curr. Biol.* 23, 1601–1605. doi:10.1016/j.cub.2013.07.030.](https://www.zotero.org/google-docs/?iJx1rw)

[Jayewardene, A. F., Gwinn, T., Hancock, D. P., Mavros, Y., and Rooney, K. B. (2014). The associations between polymorphisms in the CD36 gene, fat oxidation and cardiovascular disease risk factors in a young adult Australian population: A pilot study. *Obes. Res. Clin. Pract.* 8, e618–e621. doi:10.1016/j.orcp.2014.09.001.](https://www.zotero.org/google-docs/?iJx1rw)

[Jayewardene, A. F., Mavros, Y., Hancock, D. P., Gwinn, T., and Rooney, K. B. (2016). Associations between CD36 gene polymorphisms, fat tolerance and oral fat preference in a young-adult population. *Eur. J. Clin. Nutr.* 70, 1325–1331. doi:10.1038/ejcn.2016.132.](https://www.zotero.org/google-docs/?iJx1rw)

[Keller, K. L., Liang, L. C. H., Sakimura, J., May, D., van Belle, C., Breen, C., et al. (2012). Common Variants in the CD36 Gene Are Associated With Oral Fat Perception, Fat Preferences, and Obesity in African Americans. *Obesity* 20, 1066–1073. doi:10.1038/oby.2011.374.](https://www.zotero.org/google-docs/?iJx1rw)

[Keller, M., Liu, X., Wohland, T., Rohde, K., Gast, M.-T., Stumvoll, M., et al. (2013). TAS2R38 and Its Influence on Smoking Behavior and Glucose Homeostasis in the German Sorbs. *PLoS ONE* 8, e80512. doi:10.1371/journal.pone.0080512.](https://www.zotero.org/google-docs/?iJx1rw)

[Kirac, D., Kasimay Cakir, O., Avcilar, T., Deyneli, O., Kurtel, H., Yazici, D., et al. (2016). Effects of MC4R, FTO, and NMB gene variants to obesity, physical activity, and eating behavior phenotypes. *IUBMB Life* 68, 806–816. doi:10.1002/iub.1558.](https://www.zotero.org/google-docs/?iJx1rw)

[Knaapila, A., Hwang, L.-D., Lysenko, A., Duke, F. F., Fesi, B., Khoshnevisan, A., et al. (2012). Genetic Analysis of Chemosensory Traits in Human Twins. *Chem. Senses* 37, 869–881. doi:10.1093/chemse/bjs070.](https://www.zotero.org/google-docs/?iJx1rw)

[Landgren, S., Simms, J. A., Thelle, D. S., Strandhagen, E., Bartlett, S. E., Engel, J. A., et al. (2011). The Ghrelin Signalling System Is Involved in the Consumption of Sweets. *PLoS ONE* 6, e18170. doi:10.1371/journal.pone.0018170.](https://www.zotero.org/google-docs/?iJx1rw)

[Lee, K., Abrahamowicz, M., Leonard, G., Richer, L., Perron, M., Veillette, S., et al. (2015). Prenatal exposure to cigarette smoke interacts with OPRM1 to modulate dietary preference for fat. *J. Psychiatry Neurosci.* 40, 38–45. doi:10.1503/jpn.130263.](https://www.zotero.org/google-docs/?iJx1rw)

[Love-Gregory, L., Sherva, R., Sun, L., Wasson, J., Schappe, T., Doria, A., et al. Variants in the CD36 gene associate with the metabolic syndrome and high-density lipoprotein cholesterol. 10.](https://www.zotero.org/google-docs/?iJx1rw)

[Lunde, K., Egelandsdal, B., Skuterud, E., Mainland, J. D., Lea, T., Hersleth, M., et al. (2012). Genetic Variation of an Odorant Receptor OR7D4 and Sensory Perception of Cooked Meat Containing Androstenone. *PLoS ONE* 7, 7.](https://www.zotero.org/google-docs/?iJx1rw)

[Ma, X., Bacci, S., Mlynarski, W., Gottardo, L., Soccio, T., Menzaghi, C., et al. (2004). A common haplotype at the CD36 locus is associated with high free fatty acid levels and increased cardiovascular risk in Caucasians. *Hum. Mol. Genet.* 13, 2197–2205. doi:10.1093/hmg/ddh233.](https://www.zotero.org/google-docs/?iJx1rw)

[Melis, M., Atzori, E., Cabras, S., Zonza, A., Calò, C., Muroni, P., et al. (2013). The Gustin (CA6) Gene Polymorphism, rs2274333 (A/G), as a Mechanistic Link between PROP Tasting and Fungiform Taste Papilla Density and Maintenance. *PLOS ONE* 8, 15.](https://www.zotero.org/google-docs/?iJx1rw)

[Melo, S. V., Agnes, G., Vitolo, M. R., Mattevi, V. S., Campagnolo, P. D. B., and Almeida, S. Evaluation of the association between the TAS1R2 and TAS1R3 variants and food intake and nutritional status in children. 6.](https://www.zotero.org/google-docs/?iJx1rw)

[Menashe, I., Man, O., Lancet, D., and Gilad, Y. (2003). Different noses for different people. *Nat. Genet.* 34, 143–144. doi:10.1038/ng1160.](https://www.zotero.org/google-docs/?iJx1rw)

[Mikołajczyk-Stecyna, J., Malinowska, A. M., and Chmurzynska, A. (2017). TAS2R38 and CA6 genetic polymorphisms, frequency of bitter food intake, and blood biomarkers among elderly woman. *Appetite* 116, 57–64. doi:10.1016/j.appet.2017.04.029.](https://www.zotero.org/google-docs/?iJx1rw)

[Mrizak, I., Šerý, O., Plesnik, J., Arfa, A., Fekih, M., Bouslema, A., et al. (2015). The A allele of cluster of differentiation 36 ( *CD36* ) SNP 1761667 associates with decreased lipid taste perception in obese Tunisian women. *Br. J. Nutr.* 113, 1330–1337. doi:10.1017/S0007114515000343.](https://www.zotero.org/google-docs/?iJx1rw)

[Mulianah Daya, Dwi Ari Pujianto, Fiastuti Witjaksono, Lidwina Priliani, Jimmy Susanto, Widjaja Lukito, et al. (2019). Obesity risk and preference for high dietary fat intake are determined by FTO rs9939609 gene polymorphism in selected Indonesian adults. *Asia Pac. J. Clin. Nutr.* 28. doi:10.6133/apjcn.201903_28(1).0024.](https://www.zotero.org/google-docs/?iJx1rw)

[Park, S., Zhang, X., Lee, N. R., and Jin, H.-S. (2016). TRPV1 Gene Polymorphisms Are Associated with Type 2 Diabetes by Their Interaction with Fat Consumption in the Korean Genome Epidemiology Study. *Lifestyle Genomics* 9, 47–61. doi:10.1159/000446499.](https://www.zotero.org/google-docs/?iJx1rw)

[Pirastu, N., Kooyman, M., Traglia, M., Robino, A., Willems, S. M., Pistis, G., et al. (2015). Genome-wide association analysis on five isolated populations identifies variants of the HLA-DOA gene associated with white wine liking. *Eur. J. Hum. Genet.* 23, 1717–1722. doi:10.1038/ejhg.2015.34.](https://www.zotero.org/google-docs/?iJx1rw)

[Plesník, J., Šerý, O., Khan, A. S., Bielik, P., and Khan, N. A. (2018). The rs1527483, but not rs3212018, *CD36* polymorphism associates with linoleic acid detection and obesity in Czech young adults. *Br. J. Nutr.* 119, 472–478. doi:10.1017/S0007114517003981.](https://www.zotero.org/google-docs/?iJx1rw)

[Precone, V., Beccari, T., Stuppia, L., Baglivo, M., Paolacci, S., Manara, E., et al. Taste, olfactory and texture related genes and food choices: implications on health status. 17.](https://www.zotero.org/google-docs/?iJx1rw)

[Roos, T. R., Kulemin, N. A., Ahmetov, I. I., Lasarow, A., and Grimaldi, K. (2017). Genome-Wide Association Studies Identify 15 Genetic Markers Associated with Marmite Taste Preference. *bioRxiv*, 185629. doi:10.1101/185629.](https://www.zotero.org/google-docs/?iJx1rw)

[Yang, X., He, J., Gu, D., Hixson, J. E., Huang, J., Rao, D. C., et al. (2014). Associations of Epithelial Sodium Channel Genes With Blood Pressure Changes and Hypertension Incidence: The GenSalt Study. *Am. J. Hypertens.* 27, 1370–1376. doi:10.1093/ajh/hpu060.](https://www.zotero.org/google-docs/?iJx1rw)
