# Supplementary material for: Landscape of Variability in Chemosensory Genes Associated With Dietary Preferences in Indian Population: Analysis of 1029 Indian Genomes
Source: Front Genet. 2022 Jul 12;13:878134. doi: 10.3389/fgene.2022.878134 (PMC9315315; doi:10.3389/fgene.2022.878134)

#### Supplemental Figure 1: Distribution of chemosensory gene variants in IndiGen dataset. (A) Number of variants as SNPs, Insertion (INS), and Deletion (DEL). (B) Distribution of SNPs as their allelic variations. (C) Functional distribution of variants, and (D) distribution of exonic variants.


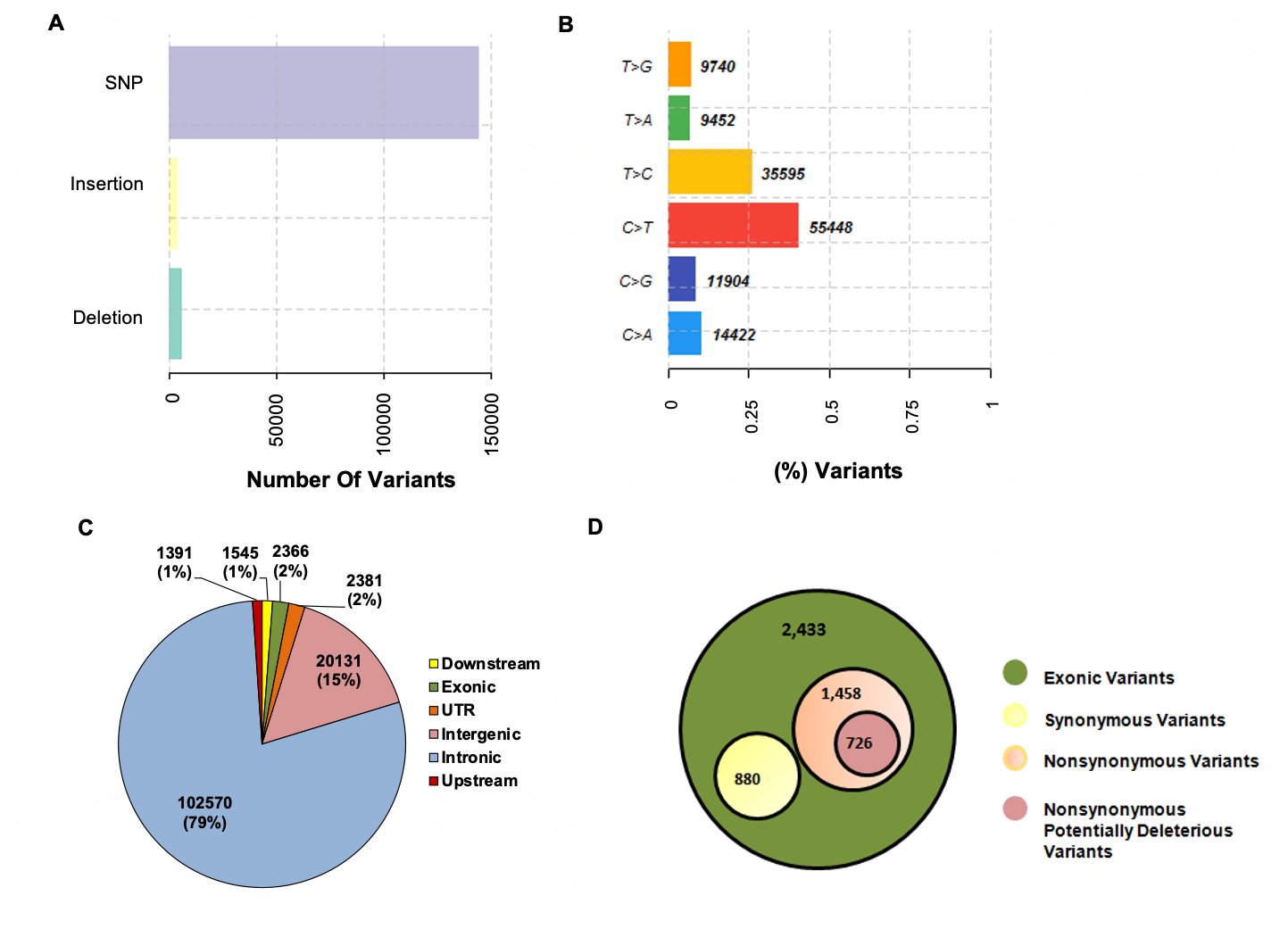


#### Supplemental Figure 2: Distribution of types of nonsynonymous variation (A) and top 10 highly exonic mutated genes within chemosensory genes.


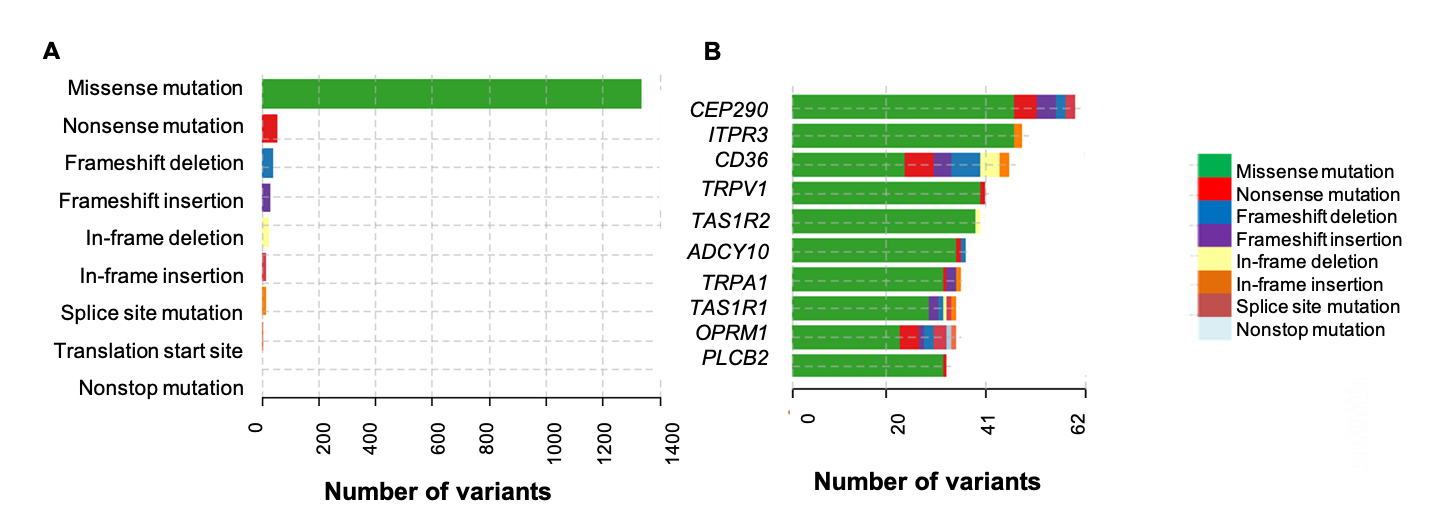


#### Supplementary Figure 3:

#### Supplemental Figure 3: (A) Venn diagram showing comparison of variants between IndiGen, 1000 genomes (excluding South-Asian (SAS) populations), and only SAS populations of 1000 genomes.


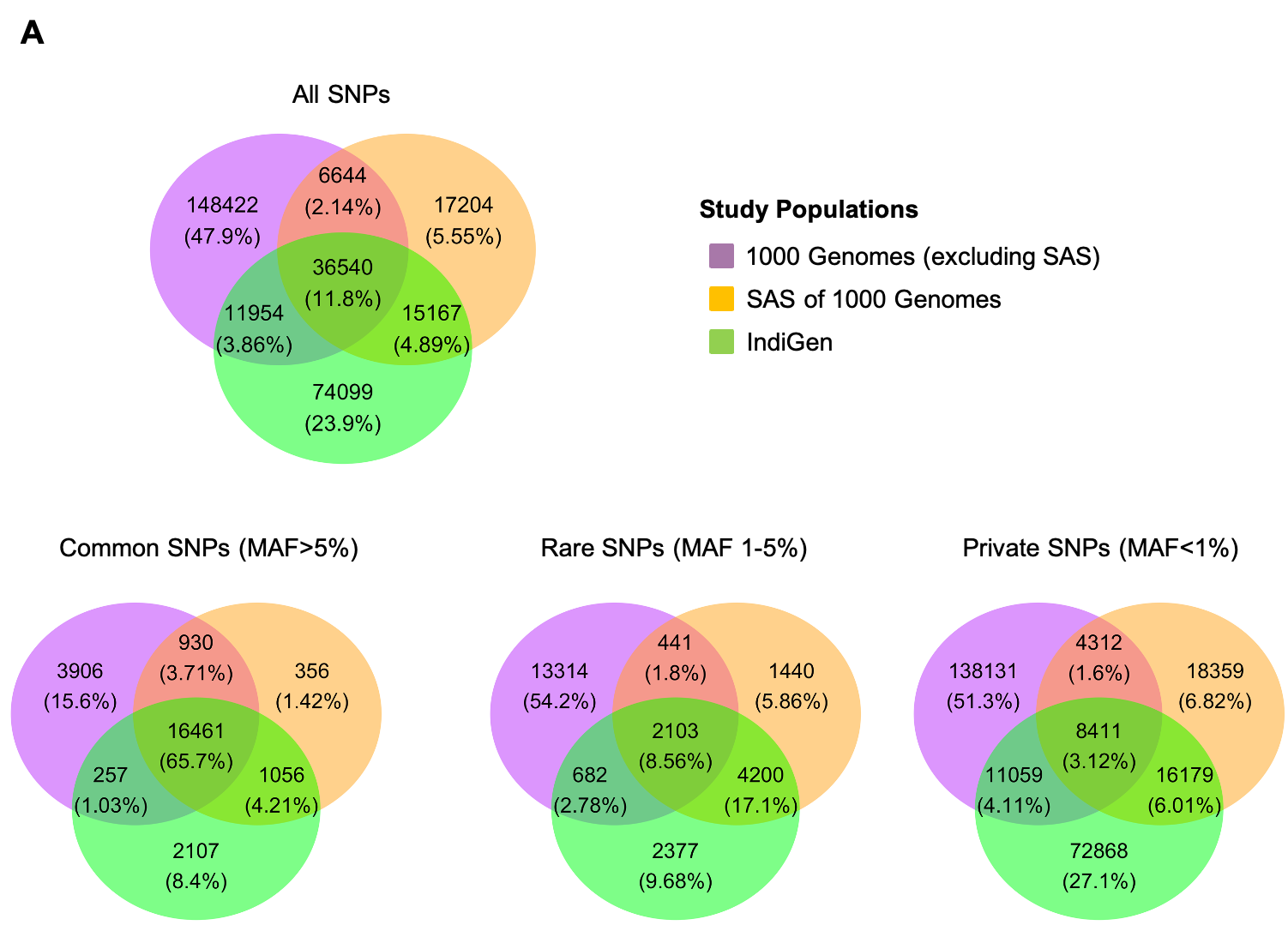


(B) Gene level comparison of variants between IndiGen, 1000 genomes (excluding SAS populations) and SAS population of 1000 genomes


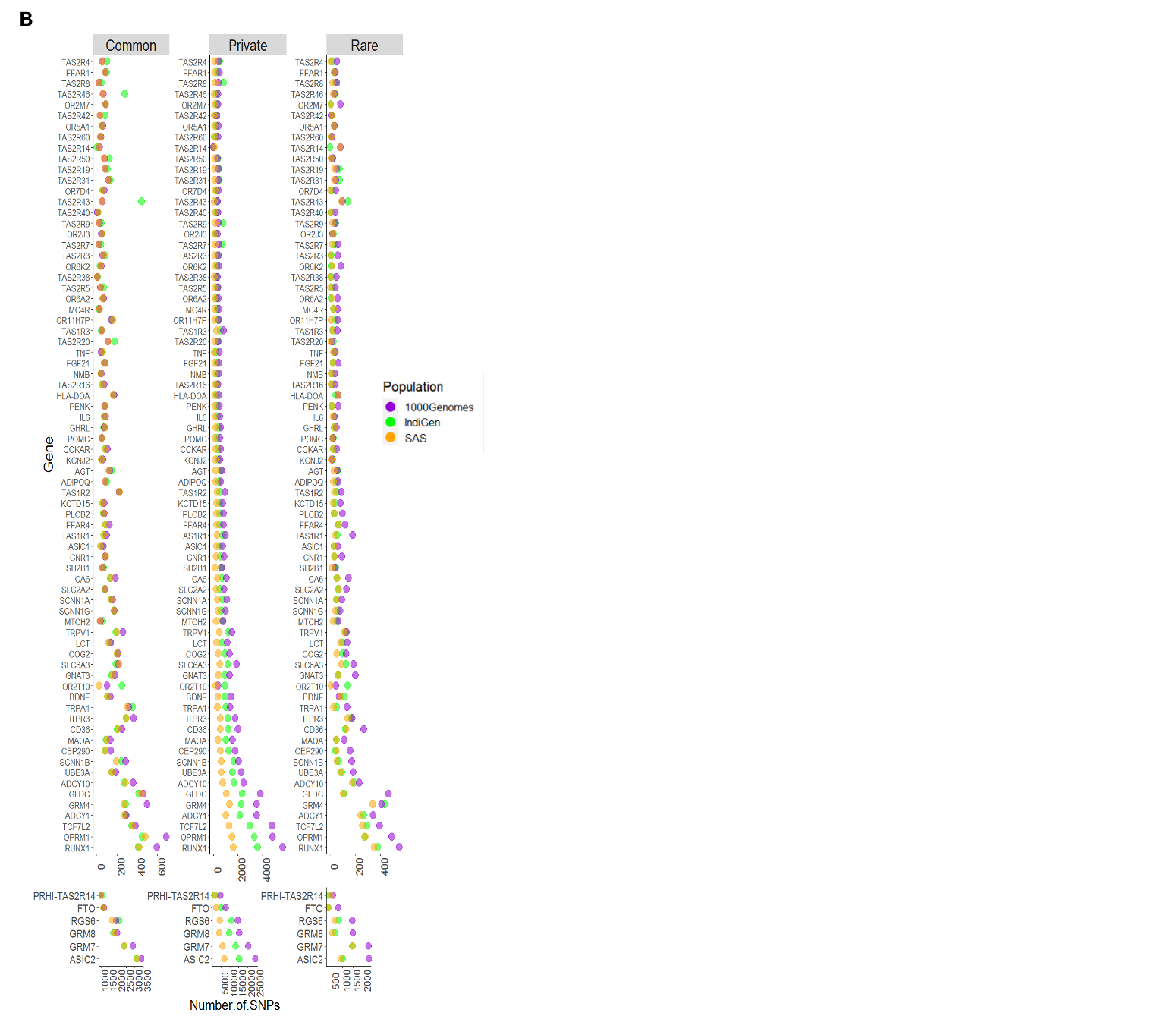


#### (C) Venn diagram showing comparison of variants between IndiGen, 1000 Genomes Project, HGSVC and SGDP


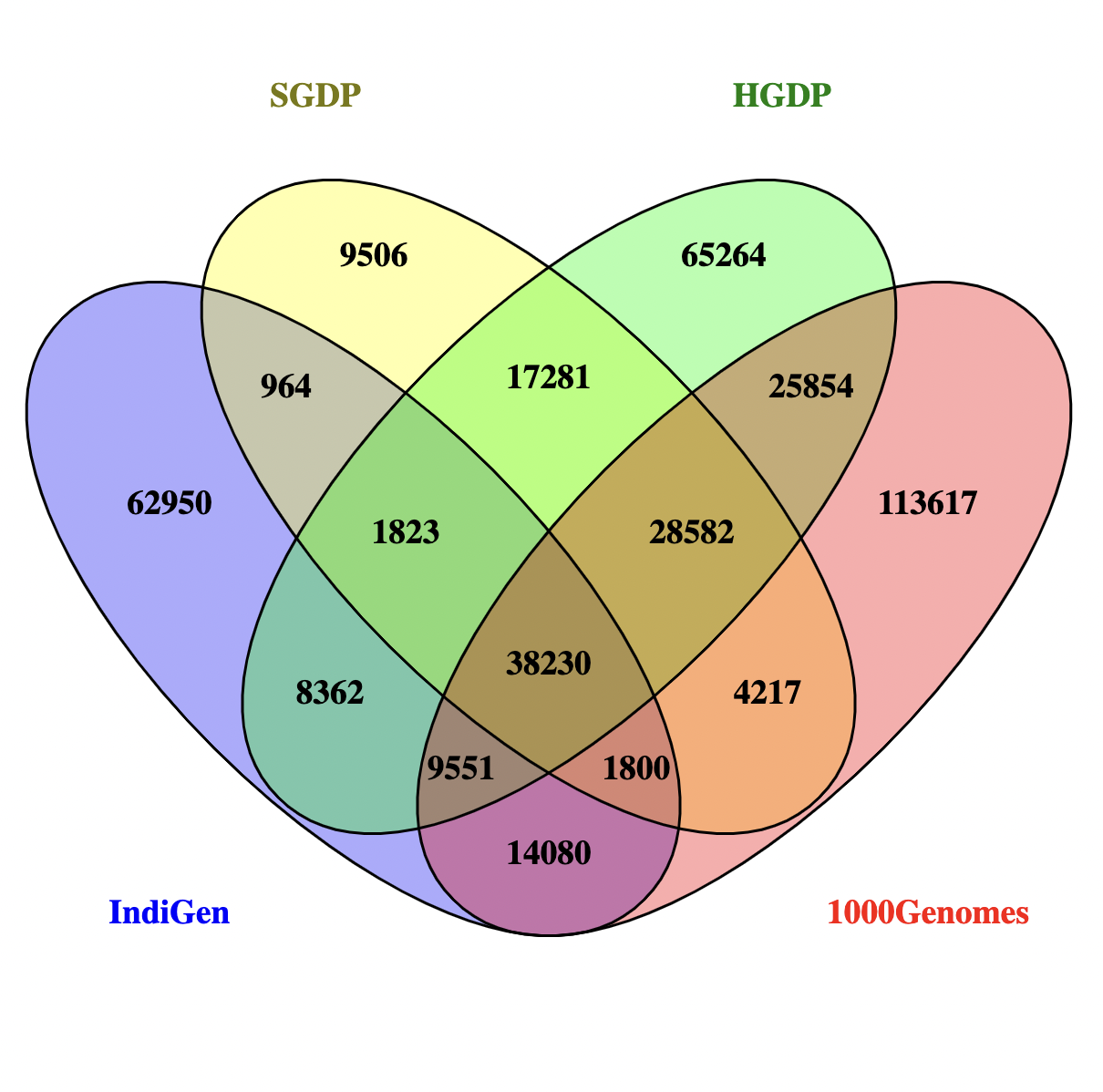


#### Supplemental Figure 4: Principal component analysis (PCA) of IndiGen samples with respect to populations from 1000 genomes and populations from Indian Genome Variation Consortium (IGVC).


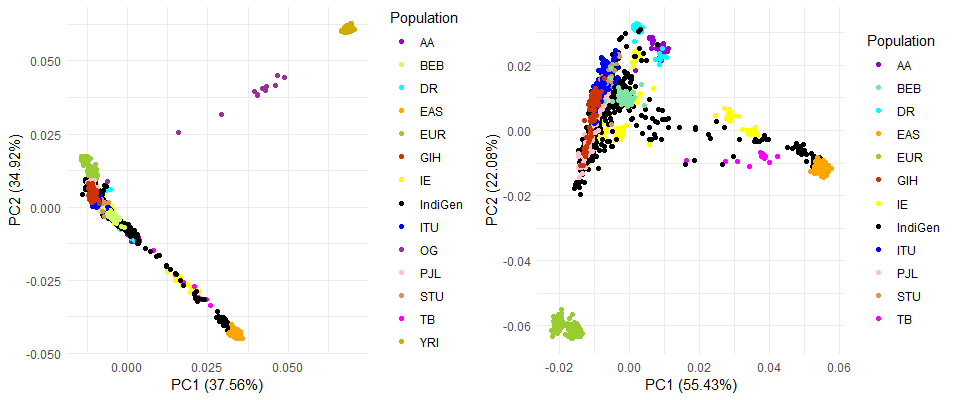


**Supplemental Figure 5:** The cross-validation error (CV-error) plot for the ADMIXTURE software analysis at different K. (A) Admixture runs for IndiGen, IGV, and 1000 Genomes combined data. (B) Admixture run for IndiGen, IGV combined data.


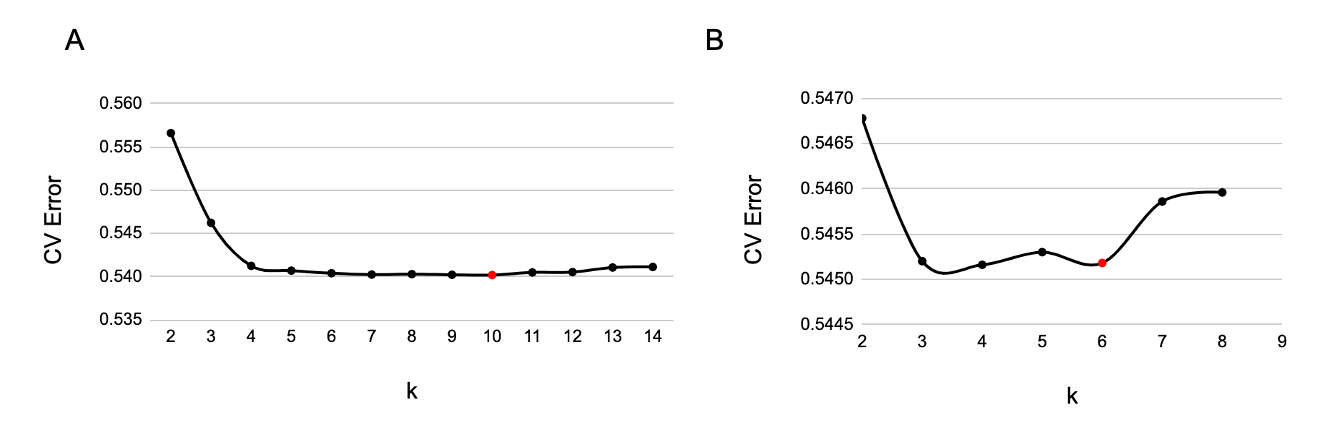


#### Supplemental Figure 6: Individual-wise admixture proportion of Indigen samples. In the plot based on admixture proportion and genetic closeness with other Indian populations, IND_1 is Tibeto-burman like, IND_2 is Indo-European like, and IND_3 is Dravidean like population.


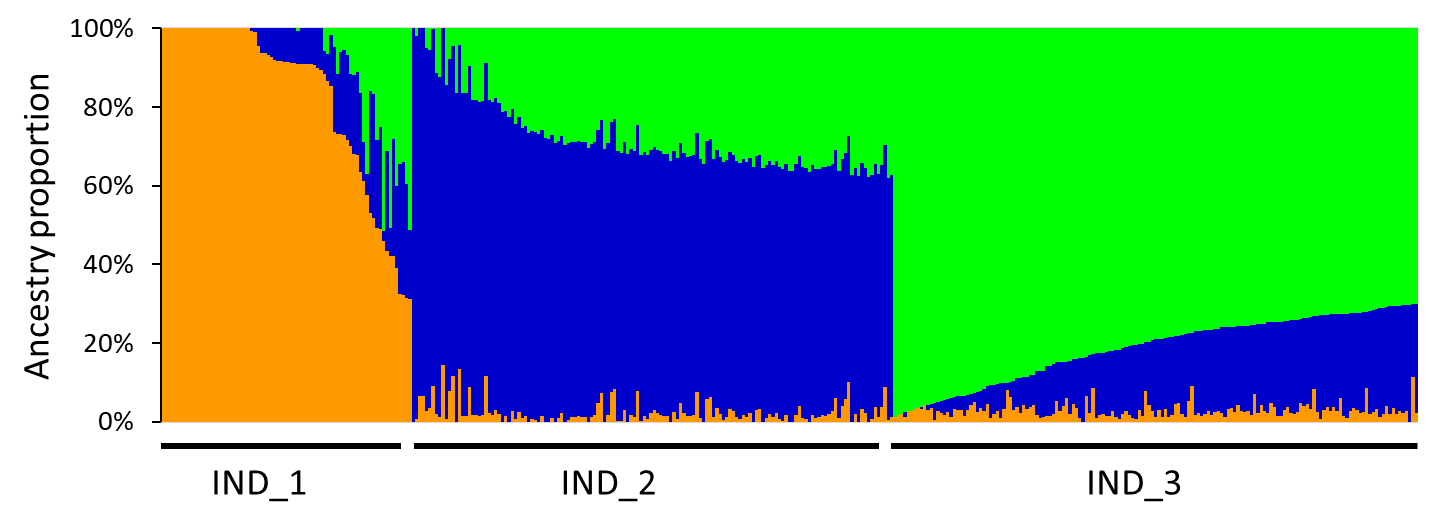


**Supplemental Figure 7:** Spatial plot of frequency distribution of (A) rs4790522 of *TRPV1* gene

in in IGVC populations of India


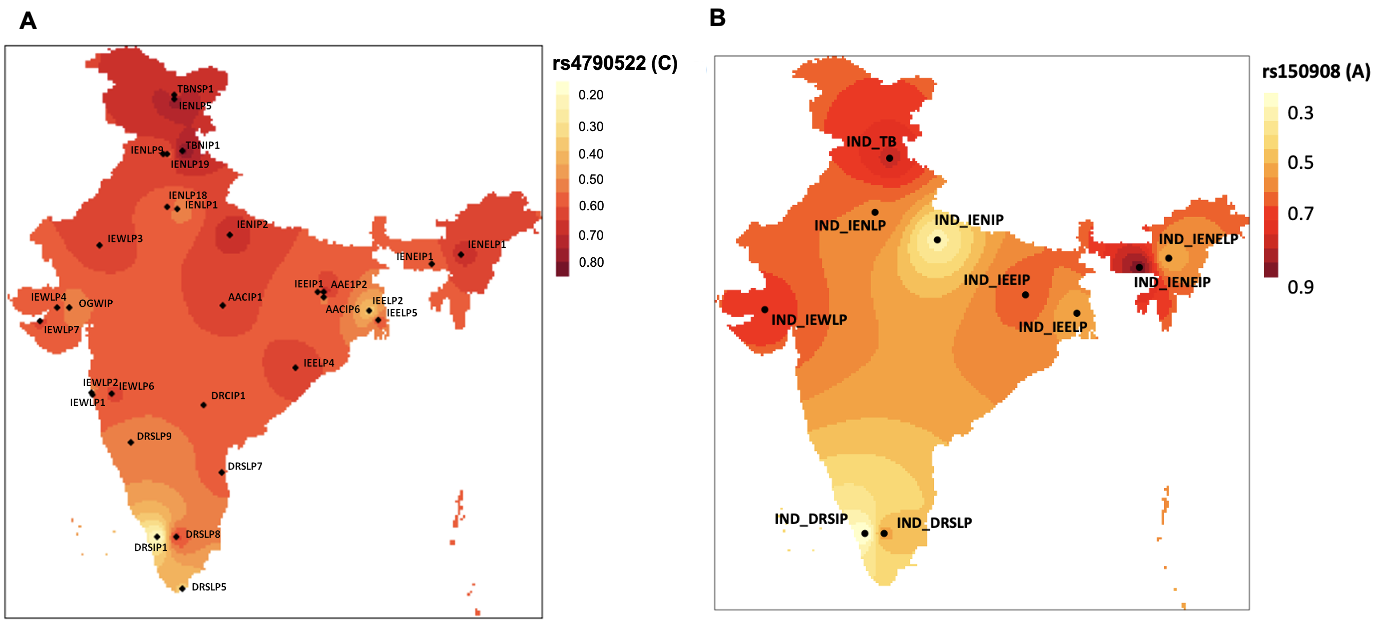

Supplement: Supplementary file 6 [file DataSheet1.docx]
